# Efficacy of acupuncture for tinnitus with anxiety: a systematic review and meta-analysis

**DOI:** 10.3389/fneur.2026.1920000

**Published:** 2026-09-22

**Authors:** Wang Peiyun, Qiu Shuaihui, Cao Yi, Yang Hao

**Affiliations:** 1 Acupuncture Department, Wuxi Second People's Hospital, Wuxi, Jiangsu, China; 2 Acupuncture Department, Wuxi Second Hospital of Traditional Chinese Medicine, Wuxi, Jiangsu, China; 3 Acupuncture Department, The Second Affiliated Hospital of Anhui University of Chinese Medicine, Hefei, Anhui, China

**Keywords:** acupuncture, anxiety states, meta-analysis, randomized controlled trials, tinnitus

## Abstract

**Background:**

Evidence suggests that acupuncture can alleviate tinnitus symptoms and improve the anxiety associated with tinnitus, but the relationship between these outcomes is unclear.

**Objectives:**

To evaluate the efficacy of acupuncture for tinnitus with anxiety using a meta-analysis.

**Methods:**

Computerized searches were conducted in nine databases: China National Knowledge Infrastructure (CNKI), Wanfang Data, VIP Chinese Science and Technology Journal Full-text Database, SinoMed, Chinese Medical Journal Full-text Database (CMAJD), PubMed, Embase, the Cochrane Library, and Web of Science. The search covered the period from the establishment of each database to January 15, 2026. Relevant randomized controlled trials meeting the inclusion and exclusion criteria were selected. The Cochrane Collaboration's recommended methods were used to assess the quality of included studies. Outcomes evaluated included the total effective rate, Tinnitus Severity Questionnaire (TEQ), Tinnitus Handicap Inventory (THI), Traditional Chinese Medicine syndrome scores (TCM syndrome scores), Hamilton Anxiety Scale (HAMA), Self-Rating Anxiety Scale (SAS), Beck Anxiety Inventory (BAI), brain-derived neurotrophic factor (BDNF), serum 5-hydroxytryptamine (5-HT), and serum gamma-aminobutyric acid (GABA) levels as outcome measures. Meta-analysis was performed using ReMan 5.3 software.

**Results:**

Twelve studies involving 908 patients were included. Meta-analysis revealed that acupuncture demonstrated superiority in improving the total effective rate for tinnitus with anxiety (RR = 1.18, 95% CI: 1.10–1.26, *P* < 0.00001) and significantly reduced TEQ scores (MD = −1.96, 95% CI: −2.47 to −1.45, Z = 7.57, *P* < 0.00001), and THI (MD = −9.11, 95% CI: −12.89 to −5.34, Z = 4.73, *P* < 0.00001), TCM syndrome scores (MD = −0.59, 95% CI: −1.13 to −0.06, Z = 2.17, *P* = 0.03 < 0.05), HAMA (MD = −2.04, 95% CI: −3.08 to −1.00, Z = 3.84, *P* = 0.0001 < 0.05) scores, SAS (MD = −3.50, 95% CI: −6.22 to −0.77, Z = 2.52, *P* = 0.01 < 0.05), BAI (MD = −8.80, 95% CI: −13.48 to −4.12, Z = 3.68, *P* = 0.0002 < 0.05) scores, but not in improving BDNF (MD = −72.27, 95% CI: −312.41 to 167.87, Z = 0.59, *P* = 0.56 > 0.05), 5-HT (MD = −22.56, 95% CI: −56.12 to 10.99, Z = 1.32, *P* = 0.19 > 0.05), and GABA (MD = 56.23, 95% CI: −46.50 to 158.96, Z = 1.07, *P* = 0.28 > 0.05) levels.

**Conclusions:**

Acupuncture treatment for tinnitus with anxiety demonstrated greater improvement than non-acupuncture approaches in enhancing the clinical total effective rate and reducing TEQ, THI, TCM syndrome scores, BAI, HAMA, and SAS scores, warranting clinical promotion. However, evidence levels for certain outcome measures (BDNF, 5-HT, and GABA) remain low, necessitating further validation through well-designed, methodologically rigorous, high-quality, large-scale randomized controlled trials.

**Systematic review registration:**

https://www.crd.york.ac.uk/PROSPERO/view/CRD42024596594; identifier: CRD42024596594.

## 1 Introduction

Tinnitus refers to the subjective perception of ringing or other sounds in the ears or head in the absence of corresponding acoustic or electrical stimuli. This auditory sensation may manifest as a single tone or multiple tones occurring simultaneously. There is currently no agreement on the pathophysiology of tinnitus. The majority of researchers think that aberrant lesions in the peripheral auditory system or changes in the synaptic structure of neurons in the auditory cortex cause improper auditory nerve discharge, which causes or exacerbates tinnitus symptoms. Additionally, some researchers believe that tinnitus is caused by specific underlying systemic factors, such as a deficiency of vitamin D3 (1). According to the 2022 JAMA Disease Survey, tinnitus affects around 740 million people globally, with a prevalence of about 14.4%. Over 120 million of them have severe tinnitus (2). Furthermore, research indicates that around 46% of people with tinnitus also have anxiety and depression, and that the degree of tinnitus is correlated with these conditions (3). Studies show that anxiety and depression are associated with lower quality of life (4). In everyday life, tinnitus is a common clinical symptom. Patients frequently feel uneasy, anxious, depressed, or afraid when the condition worsens since they are unable to manage or avoid it. A vicious loop known as “tinnitus-anxiety state-amplified tinnitus” can result from negative emotions making tinnitus worse (5). Tinnitus coupled by anxiety may be treated with acupuncture, a unique technique in traditional Chinese medicine (6). Numerous scholars have conducted original research on acupuncture for tinnitus with anxiety (7–10), but systematic reviews or meta-analyses remain scarce. In order to thoroughly evaluate the effectiveness of acupuncture for tinnitus with anxiety and offer evidence-based treatment recommendations, this study does a systematic review and meta-analysis of randomized controlled trials (RCTs). The study has been filed on the PROSPERO website with registration number CRD42024596594 and will closely follow the rules specified in the Protocol Registration and Systematic Review and Meta-Analysis Protocol Statement (PRISMA-P) (11).

## 2 Data and methods

### 2.1 Literature inclusion and exclusion criteria

(1) Study type: randomized controlled trials (RCTs) conducted in Chinese and English, regardless of blinding or allocation concealment. (2) Study population: individuals with anxiety and tinnitus as their main symptoms. Comparability must be guaranteed, but there are no limitations on patient source, age, gender, diagnostic level, or Traditional Chinese Medicine symptom pattern. Note: studies that measure anxiety-related indicators as a key outcome in addition to primarily assessing the effectiveness of acupuncture for tinnitus should be included. (3) Interventions: the treatment group received conventional acupuncture therapy, including acupuncture alone or combined with other Chinese or Western medical therapies. The control group received sham acupuncture or Western medication. (4) Outcomes: primary outcomes included total effective rate, Tinnitus Severity Rating Scale (TEQ), Tinnitus Handicap Inventory (THI), and Traditional Chinese Medicine (TCM) syndrome scores. Secondary outcomes included the Hamilton Anxiety Rating Scale (HAMA), Self-Rating Anxiety Scale (SAS), Beck Anxiety Inventory (BAI), and levels of brain-derived neurotrophic factor (BDNF), serum 5-hydroxytryptamine (5-HT), and serum gamma-aminobutyric acid (GABA). (5) Exclusion Criteria: Non-RCT clinical research literature; Studies employing acupuncture intervention but failing to report anxiety-related indicators; Duplicate publications; Full-text unavailability or missing data.

### 2.2 Literature search strategy

Comprehensive searches were conducted in nine databases: China National Knowledge Infrastructure (CNKI), Wanfang Data, VIP Chinese Science & Technology Journal Full-text Database, SinoMed, Chinese Medical Journal Full-text Database (CMAJD), PubMed, Embase, Cochrane Library, and Web of Science. The search period ran from the creation date of each database until January 15, 2026. Chinese search terms: “针” or “针刺” or “针灸” or “针法” and “焦虑” or “焦虑状态” and “耳鸣” or ““主观性耳鸣” or ““原发性耳鸣” or “神经性耳鸣”; English search terms: “Acupuncture” or “Pharmacopuncture” and “Anxiety” or “Anxiousness” or “Angst” or “Nervousness” or “Hypervigilance” and “Tinnitus.”

Chinese search query (using WanFang as an example): 题名或关键词: (耳鸣) and 题名或关键词: (耳鸣) and 题名或关键词: (针刺 or 针灸 or 针法).

English search query (using PubMed as an example): (“acupunctural”[All Fields] OR “acupuncture”[MeSH Terms] OR “acupuncture”[All Fields] OR “acupuncture therapy”[MeSH Terms] OR (“acupuncture”[All Fields] AND “therapy”[All Fields]) OR “acupuncture therapy”[All Fields] OR “acupuncture s”[All Fields] OR “acupunctured”[All Fields] OR “acupunctures”[All Fields] OR “acupuncturing”[All Fields] OR (“acupuncture”[MeSH Terms] OR “acupuncture”[All Fields] OR “pharmacopuncture”[All Fields] OR “pharmacopunctures”[All Fields])) AND (“anxiety”[MeSH Terms] OR “anxiety”[All Fields] OR “anxieties”[All Fields] OR “anxiety s”[All Fields] OR (“anxiety”[MeSH Terms] OR “anxiety”[All Fields] OR “anxiousness”[All Fields]) OR (“anxiety s”[All Fields] OR “anxiety”[MeSH Terms] OR “anxiety”[All Fields] OR “anxiety”[All Fields]) OR (“anxiety”[MeSH Terms] OR “anxiety”[All Fields] OR “nervousness”[All Fields]) OR (“anxiety”[MeSH Terms] OR “anxiety”[All Fields] OR “hypervigilance”[All Fields] OR “hypervigilant”[All Fields])) AND (“tinnitus”[MeSH Terms] OR “tinnitus”[All Fields]).

### 2.3 Literature screening and data extraction

Two researchers independently examined each title and abstract for initial screening based on the inclusion and exclusion criteria. After that, they carefully selected relevant literature based on the criteria and carried out a thorough full-text evaluation of the screened studies. Both researchers conducted a cross-check; any differences in the extracted literature needed to be discussed with a third researcher in order to be resolved. Ultimately, an information database was created by combining the findings from the literature. The original authors were contacted in order to gather any missing data from the included study reports. To check the authenticity and completeness of the data, the study was rejected if missing data could not be obtained. Researcher names, publication year, sample size, participant baseline characteristics, intervention measures, and outcome measures were among the extracted and validated data. The material was managed using EndNote X9.1 software (Philadelphia, PA, USA: Clarivate Analytics, 2019).

### 2.4 Literature quality assessment (revised)

Two researchers independently assessed and cross-checked the included studies using the revised Cochrane tool (RoB2). The assessment covered five domains—randomization process, intended intervention, missing outcome data, outcome measurement, and selective reporting of results—and evaluated each domain according to its algorithm, culminating in an overall risk assessment. Risk levels were categorized as low, high, or unclear. A third researcher was consulted to settle inconsistencies in studies that were challenging to evaluate.

### 2.5 Statistical analysis

Meta-analysis was performed using RevMan 5.3 software (Copenhagen: The Nordic Cochrane Collaboration, 2014). For continuous data with consistent units, the weighted mean difference (WMD) was used as the reference score for TEQ, THI, TCM syndrome scores, HAMA, SAS, BAI, and 5-HT. For continuous variables with inconsistent units, data were first converted and then WMD was used as the reference for scoring BDNF, and GABA; For categorical data, relative risk (RR) was used as the reference for scoring the overall effective rate; All effect sizes were expressed with 95% confidence intervals (95% CI), with P < 0.05 indicating statistically significant differences. *I*^2^ and *P*-values were used to evaluate heterogeneity among the included studies. A fixed-effects model was employed for analysis if *P* ≧ 0.1 and *I*^2^ < 50%, indicating acceptable heterogeneity; on the other hand, a random-effects model was warranted if *P* < 0.1 and *I*^2^≥ 50%, indicating substantial heterogeneity. The visual evaluation of publication bias was done using funnel plots. Sensitivity analysis was carried out by gradually eliminating individual studies; the outcomes were stable and did not significantly alter.

### 2.6 Grading the quality of evidence

The Recommended Scoring for Evaluation, Development, and Assessment (Grade) approach (12) was used to evaluate the quality of the evidence. A number of issues, such as possible publication bias, inconsistency, inhomogeneity, imprecision, and risk of bias, might cause evidence that was previously evaluated as high-quality in RCTs to be downgraded. After then, the quality of the evidence is evaluated and divided into four categories: high, moderate, low, and very low.

## 3 Results

### 3.1 Literature search

949 documents were found in the first search. 288 duplicate documents were eliminated using EndNote 20 reference management software. After preliminary screening based on titles and abstracts, 434 papers that did not fulfill the criteria were eliminated. Following full-text screening, 215 documents were eliminated. In the end, Figure 1 displays 12 documents (11–22).

**Figure 1:**
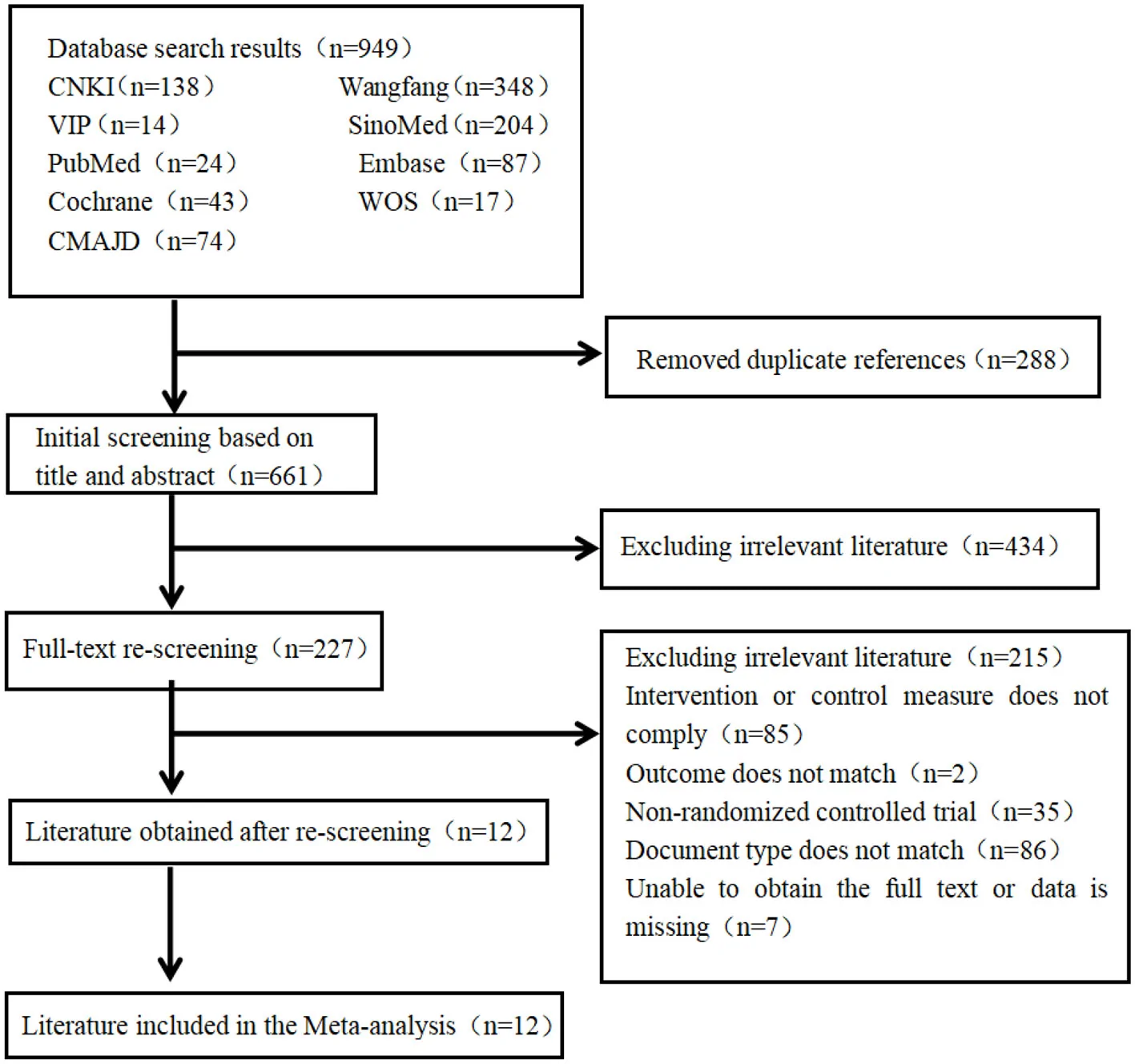
Literature search flowchart.

### 3.2 Basic characteristics of included literature

Twelve publications (13–24) with a total of 908 subjects-−455 in the treatment group and 453 in the control group—were included in this study; ten articles (13–21, 24) were written in Chinese, and two articles (22, 23) were written in English. Ten clinical trials were carried out in China (13–21, 24), one in Brazil (22), and one in Iran (23). All studies reported comparable baseline characteristics across groups. Three studies (22–24) compared conventional acupuncture with sham acupuncture, two studies (13, 20) compared conventional acupuncture with Western medicine alone, four studies (14, 17–19) compared conventional acupuncture combined with Chinese herbal medicine vs. Chinese herbal medicine alone, one study (15) compared conventional acupuncture combined with Western medicine vs. Western medicine alone, one study (16) compared conventional acupuncture combined with tuina massage vs. tuina massage alone, and one study (21) compared special acupoint acupuncture combined with Western medicine to Western medicine combined with conventional acupuncture. Nine studies (13–21) included the total effective rate among outcome measures, and seven studies (13–15, 18–20) included TEQ, nine studies (15–22, 24) included THI, four studies (16, 17, 19, 21) included TCM syndrome scores, four studies (14, 20, 22, 24) included HAMA, two studies (15, 17) included SAS, two studies (16, 22) included BAI, two studies (13, 24) included BDNF, two studies (21, 24) included 5-HT, two studies (21, 24) included GABA. Refer to Table 1.

**Table 1:** Basic characteristics of included studies.

Included studies	Sample size (cases)		Gender (male/female, cases)		Age $\bar{x}$ ±s, years)		Duration of illness ($\bar{x}$ ±s, months/years)		Intervention		Outcome measures
	T	C	T	C	T	C	T	C	T	C	
Li et al. (13)	41	44	16/25	18/26	42 ± 12	44 ± 11	8.7 ± 4.3	7.8 ± 3.5	Acupuncture	Western Medicine	①, ②, ⑧
Wang et al. (14)	30	30	13/17	14/16	65.38 ± 5.19	66.21 ± 8.13	3.22 ± 0.94	3.61 ± 1.32	Acupuncture + Chinese Herbal Medicine	Chinese Herbal Medicine	①, ②, ⑤
Chen et al. (15)	63	63	41/22	39/24	50.36 ± 7.33	53.6 ± 9.6	25.3 ± 3.4	21.6 ± 4.6	Acupuncture + Western Medicine	Western Medicine	①, ②, ③, ⑥
Luo et al. (16)	41	41	23/18	20/21	61.68 ± 5.24	63.24 ± 4.85	27.55 ± 11.14	26.36 ± 11.03	Acupuncture + Massage	Tuina	①, ③, ④, ⑦
Dong et al. (17)	30	30	16/14	14/16	39.73 ± 12.35	39.03 ± 11.7	2.75 ± 0.5	2.78 ± 0.5	Acupuncture + Chinese Herbal Medicine	Chinese Herbal Medicine	①, ③, ④, ⑥
Zheng and Shen (18)	51	51	25/26	23/28	45.12 ± 13.26	46.05 ± 12.17	4.42 ± 1.13	4.67 ± 1.28	Acupuncture + Chinese Herbal Medicine	Chinese Herbal Medicine	①, ②
Sun (19)	44	44	17/27	16/28	48.59 ± 10.15	48.07 ± 10.27	15.39 ± 8.35	15.18 ± 7.26	Acupuncture + Chinese Herbal Medicine	Chinese Herbal Medicine	①, ②, ③, ④
Yao et al. (20)	30	30	15/16	13/17	35.2 ± 6.2	35.9 ± 6.8	19.6 ± 4.2	17.9 ± 3	Acupuncture	Western Medicine	①, ②, ③, ⑤
Liu et al. (21)	38	38	17/21	15/23	49.64 ± 8.71	50.32 ± 8.46	1.22 ± 0.35	1.31 ± 0.37	Special Acupoints + Conventional Acupuncture + Western Medication	Western Medicine + Conventional acupuncture	①, ③, ④, ⑨, ⑩
Laureano et al. (22)	30	27	15/15	4/23	46.23 ± 9.94	44.33 ± 12.74	—	—	Acupuncture	Sham Acupuncture	③, ⑤, ⑦
Rogha et al. (23)	27	27	14/13	14/13	45.6 ± 13.55	49.2 ± 14.3	—	—	Acupuncture	Sham Acupuncture	②
Zhang et al. (24)	30	28	12/18	10/18	46.6 ± 11.26	43.75 ± 13.14	2.41 ± 0.55	3.06 ± 0.87	Acupuncture	Sham Acupuncture	③, ⑤, ⑧, ⑨, ⑩

T, Treatment group; C, Control group. Outcome measures: ① Overall response rate; ② TEQ; ③ THI; ④ TCM syndrome scores; ⑤ HAMA; ⑥ SAS; ⑦ BAI; ⑧ BDNF; ⑨ 5-HT; ⑩ GABA.

Among the interventions, 11 studies (11, 12, 14–21) used conventional acupuncture, and one study (15) used magnetic Wenmei acupuncture along meridians combined with conventional acupuncture. One study (23) didn't mention the duration of a single treatment, while the other 11 studies (13–22, 24) did; among these, nine studies (13–21) reported that a single treatment took 30 min, and two studies (22, 24) reported 20 min. The treatment periods ranged from 20 to 42 days. Specifically, two studies (21, 23) required 10 sessions, two studies (13, 22) required 12 sessions, two studies (15, 24) required 18 sessions, four studies (14, 17, 18, 20) required 20 sessions, and two studies (16, 19) required 30 sessions. Out of the 12 studies (13–24), only three studies (15, 23, 24) adjusted the acupuncture points based on syndrome differentiation. See Table 2.

**Table 2:** Characteristics of interventions in the included studies.

Included studies	Acupuncture method	Single treatment time (minutes)	Number of treatments	Treatment process (Days)	Specific acupuncture points
Li et al. (13)	Regular acupuncture	30	12	28	Affected side: Zhongzhu (TE 3), Tinghui (GB 2), Yifeng (TE 17);Shuigou (GV 26), Yintang (EX-HN 3), Shenting (GV 24), Baihui (GV 20), Fengfu (GV 16), Dazhui (GV 14)
Wang et al. (14)	Regular acupuncture	30	20	28	Affected side: Tinggong (SI 19), Tinghui (GB 2), Wangu (GB 12), Fengchi (GB 20); Bilateral: Hegu (LI 4), Taichong (LR 3)
Chen et al. (15)	Magnetic round plum needle tapping along the meridians +Regular acupuncture	30	18	21	Bilateral: Yifeng (TE 17), Tinghui (GB 2). Syndrome differentiation points:Hyperactive liver and gallbladder fire add Xingjian (LR 2), Xiaxi (GB 43);Weak spleen and stomach add Pishu (BL 20), Zusanli (ST 36);Phlegm-heat accumulation add Fenglong (ST 40), Neiting (ST 44);Kidney essence deficiency add Shenshu (BL 23), Taixi (KI 3);Exogenous wind pathogen add Fengchi (GB 20), Waiguan (TE 5);Hyperactive liver yang add Taichong (LR 3), Taixi (KI 3)
Luo et al. (16)	Regular acupuncture	30	30	36	Affected side: Baihui (GV 20), Ermen (TE 21), Yifeng (TE 17), Tinghui (GB 2); Bilateral: Neiguan (PC 6), Zusanli (ST 36), Taichong (LR 3), Tianshu (ST 25), Sanyinjiao (SP 6), Qihai (CV 6)
Dong et al. (17)	Regular acupuncture	30	20	28	Affected side: Erheliao (TE 22), Tinggong (SI 19), Tinghui (GB 2), Shuaigu (GB 8), Yifeng (TE 17), Baihui (GV 20), Sishencong (EX-HN 1), Yintang (EX-HN 3); Bilateral: Hegu (LI 4), Zhongzhu (TE 3), Waiguan (TE 5), Zhigou (TE 6)
Zheng and Shen (18)	Regular acupuncture	30	20	28	Ermen (TE 21), Tinggong (SI 19), Tinghui (GB 2), Yifeng (TE 17), Fengchi (GB 20), Shenshu (BL 23), Taixi (KI 3)
Sun (19)	Regular acupuncture	30	30	42	Ermen (TE 21), Tinggong (SI 19), Tinghui (GB 2), Yifeng (TE 17), Zhongzhu (TE 3), Guanyuan (CV 4), Shenshu (BL 23), Taixi (KI 3)
Yao et al. (20)	Regular acupuncture	30	20	28	Affected side: Tinggong (SI 19), Tinghui (GB 2), Yifeng (TE 17); Bilateral: Zhongzhu (TE 3), Xiaxi (GB 43), Shenmen (HT 7), Neiguan (PC 6), Taichong (LR 3)
Liu et al. (21)	Regular acupuncture	30	10	20	Bilateral: Ermen (TE 21), Tinggong (SI 19), Tinghui (GB 2), Yifeng (TE 17), Zhongzhu (TE 3), Xiaxi (GB 43), Qihai (CV 6), Zusanli (ST 36), Pishu (BL 20)
Laureano et al. (22)	Regular acupuncture	20	12	42	Baihui (GV 20), Yifeng (TE 17), Shuaigu (GB 8), Tinggong (SI 19), Tinghui (GB 2), Ermen (TE 21), Lianquan (CV 23), Fengchi (GB 20), Yemen (TE 2), Waiguan (TE 5), Qiangu (SI 66), Xiaxi (GB 43), Zulinqi (GB 41), Mingmen (GV 4), Taixi (KI 3), Shenshu (BL 23), Danshu (BL 19), Ganshu (BL 18), Guanyuan (CV 4)
Rogha et al. (23)	Regular acupuncture	Not mentioned	10	20	Yifeng (TE 17), Tinghui (GB 2), Tinggong (SI 19), Ermen (TE 21). Syndrome differentiation points: Zhongzhu (TE 3), Waiguan (TE 5), Shuaigu (GB 8), Fengchi (GB 20), Xiaxi (GB 43), Xingjian (LR 2); Hegu (LI 4), Yinlingquan (SP 9), Fenglong (ST 40), Pishu (BL 20), Fengchi (GB 20), Shuifen (CV 9), Zhongwan (CV 12), Waiguan (TE 5); Sanyinjiao (SP 6), Yinxi (HT 6), Shenshu (BL 23), Taixi (KI 3), Fuliu (KI 7), Taichong (LR 3), Mingmen (GV 4), Guanyuan (CV 4); Taiyuan (LU 9), Feishu (BL 13), Tianyou (TE 16), Baihui (GV 20), Qihai (CV 6), Danzhong (CV 17); Sanyinjiao (SP 6), Xinshu (BL 15), Neiguan (PC 6), Baihui (GV 20), Jujue (CV 14)
Zhang et al. (24)	Regular acupuncture	20	18	42	Affected side: Tinggong (SI 19), Tinghui (GB 2), Ermen (TE 21), Yifeng (TE 17); Bilateral: Daling (PC 7), Laogong (PC 8);Baihui (GV 20), Shenting (GV 24). Syndrome differentiation points: Weak spleen and stomach add Zusanli (ST 36), Qihai (CV 6); Kidney essence deficiency add Guanyuan (CV 4), Sanyinjiao (SP 6); Phlegm-dampness retention add Fenglong (ST 40), Zusanli (ST 36); Liver qi stagnation add Hegu (LI 4), Taichong (LR 3)

### 3.3 Quality assessment of included literature

Quality assessment was conducted on 12 included studies. Randomization process: Nine studies (13–15, 18–22, 24) used random number tables to generate random sequences, all rated as “low risk”; the remaining three studies (16, 17, 23) only described random allocation without specifying the method, rated as “unknown risk.” Deviation from intended intervention: Three studies (13, 22, 24) employed randomized controlled single-blind trial designs, and two studies (13, 24) specifically noted the use of envelope blinding, both rated as “low risk”; the remaining nine studies (14–21, 23) did not mention blinding implementation. However, due to the specificity of acupuncture treatment, it is difficult to implement blinding in the trials, so it is assessed as “uncertain risk.” Missing outcome data: all trials provided complete outcome data, eliminating missing outcome data concerns, while three studies (13, 22, 24) reported case dropouts. All were so classified as “low risk.” Measuring the outcome: since all 12 of the included studies (13–24) measured outcomes using suitable techniques, they were all classified as “low risk.” Selection of outcome reporting: eight studies (13–16, 20, 22–24) published clinical trial procedures, among which five (13, 15, 22–24) provide specific registration numbers and were rated as “low risk”; the other four (17–19, 21) did not provide this information and were rated as “risk unknown.” Refer to Figure 2.

**Figure 2:**
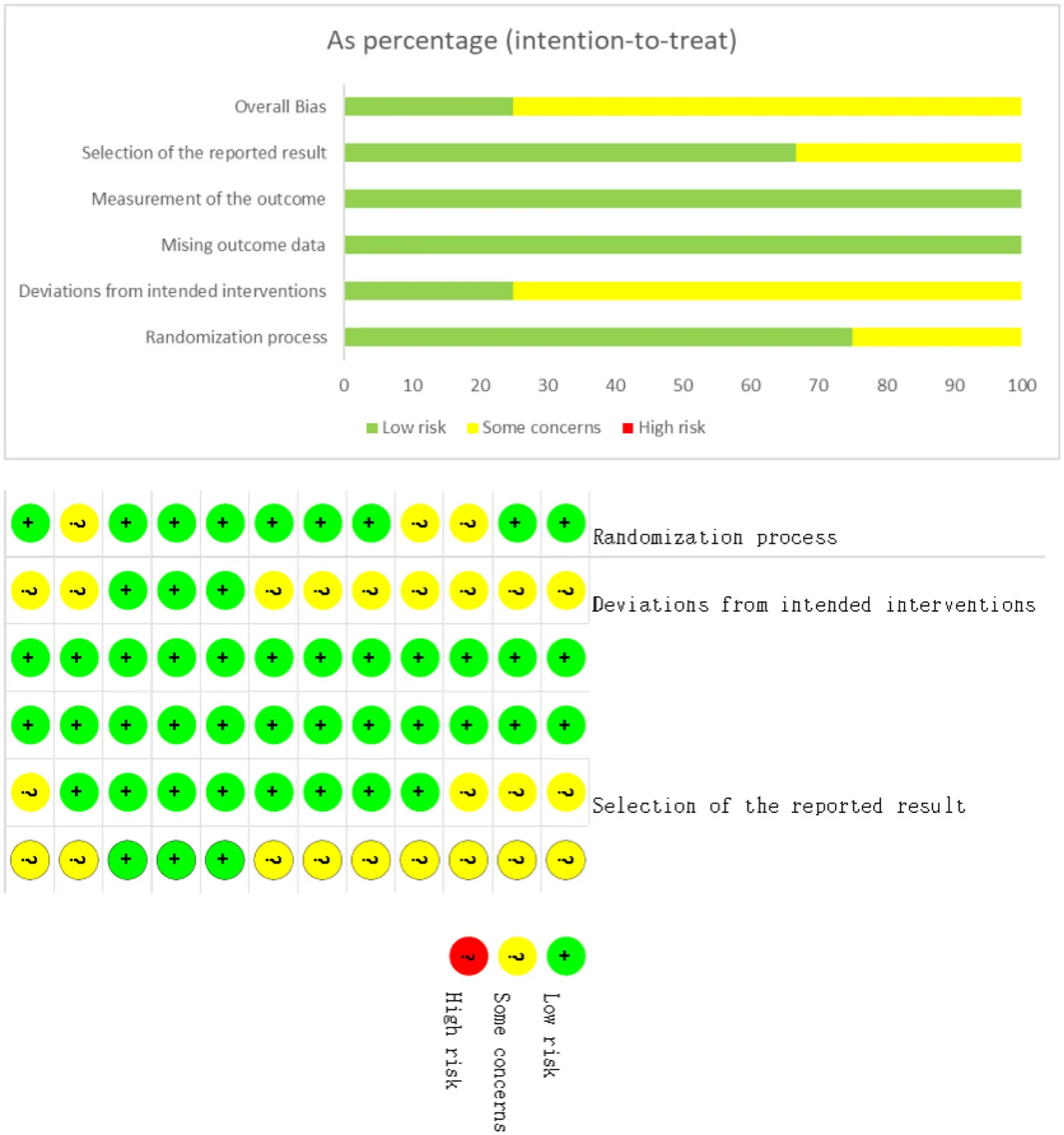
Quality assessment of included studies.

### 3.4 Meta-analysis of included literature

#### 3.4.1 Overall efficacy rate

Nine (13–21) of the 12 included trials reported clinical effectiveness. There was enough similarity between the approaches employed to assess the overall efficacy rate on various scales to permit pooled analysis. The following criteria were used: cure was defined as the resolution of tinnitus symptoms; significant effect was defined as a marked improvement in tinnitus symptoms with a ≧ 2-grade reduction in severity; effective response was defined as an improvement in tinnitus symptoms with a 1-grade reduction in severity; failure to meet any of these criteria was classified as ineffective. 329 successful responses (89.4%) among 368 patients in the treatment group and 281 effective responses (75.7%) among 371 patients in the control group were found when the overall efficacy rates from these nine studies were analyzed. Results reveal that the overall efficacy rate in the treatment group was considerably greater than that in the control group (RR = 1.18, 95% CI: 1.10–1.26, *P* < 0.00001, *I*^2^ = 0%), suggesting that acupuncture treatment for tinnitus with anxiety generates superior clinical efficacy compared to the control group. Refer to Figure 3.

**Figure 3:**
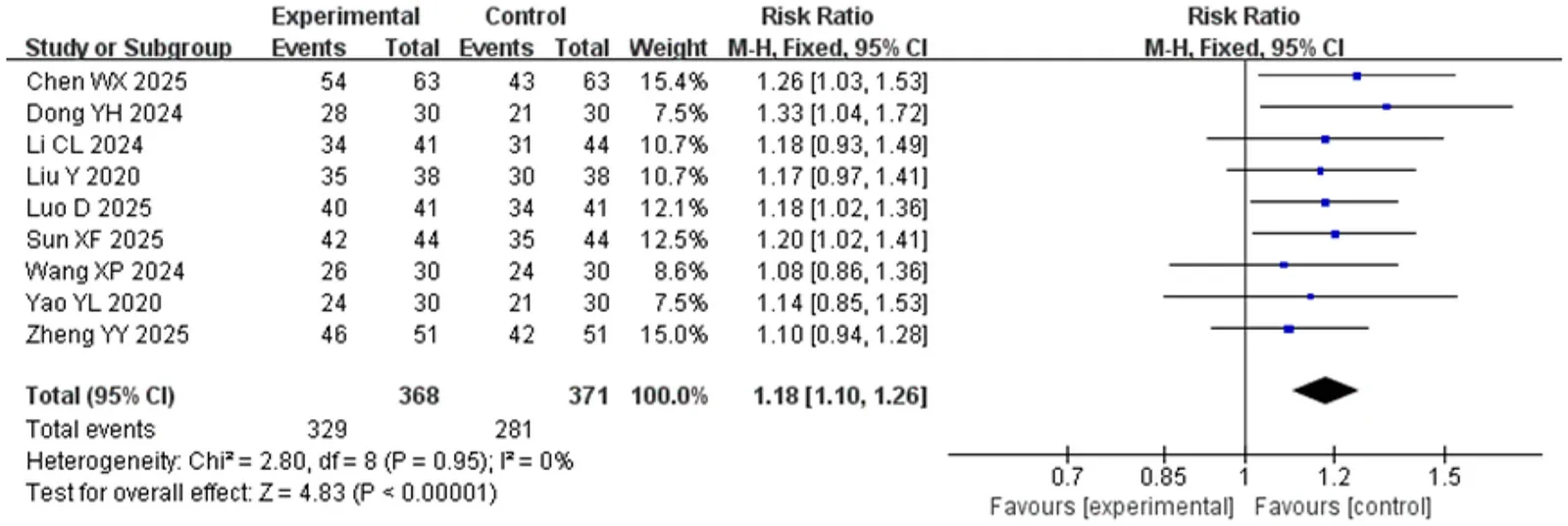
Forest plot of meta-analysis for overall response rate.

#### 3.4.2 Tinnitus severity assessment scale (TEQ) scores

Seven (13–15, 18–20) of the 12 included trials reported TEQ scores before and after treatment. Results demonstrated that the treatment group considerably outperformed the control group in reducing TEQ scores (MD = −1.96, 95% CI: −2.47 to −1.45, *P* < 0.00001, *I*^2^= 48%). Refer to Figure 4. Sensitivity analysis shows the results are robust.

**Figure 4:**
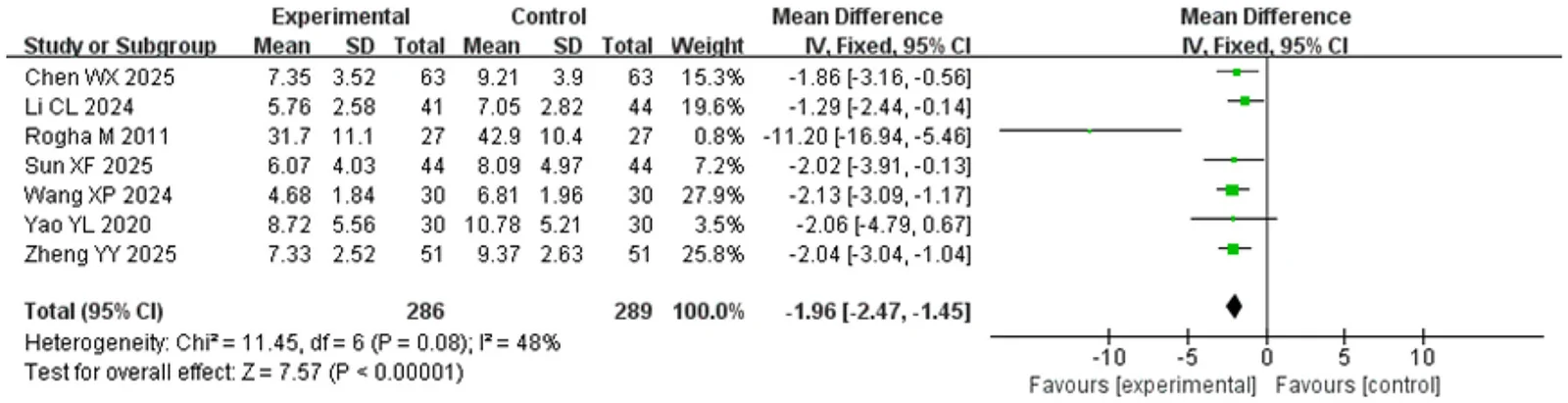
Meta-analysis forest plot of TEQ scores for acupuncture treatment of tinnitus with anxiety.

#### 3.4.3 Tinnitus handicap inventory (THI) scores

Nine (15–22, 24) of the 12 included trials reported THI scores before and after treatment. Results demonstrated that the treatment group considerably outperformed the control group in reducing THI scores (MD = −9.11, 95% CI: −12.89 to −5.34, *P* < 0.00001, *I*^2^ = 95%). Refer to Figure 5. Sensitivity analysis shows the results are robust.

**Figure 5:**
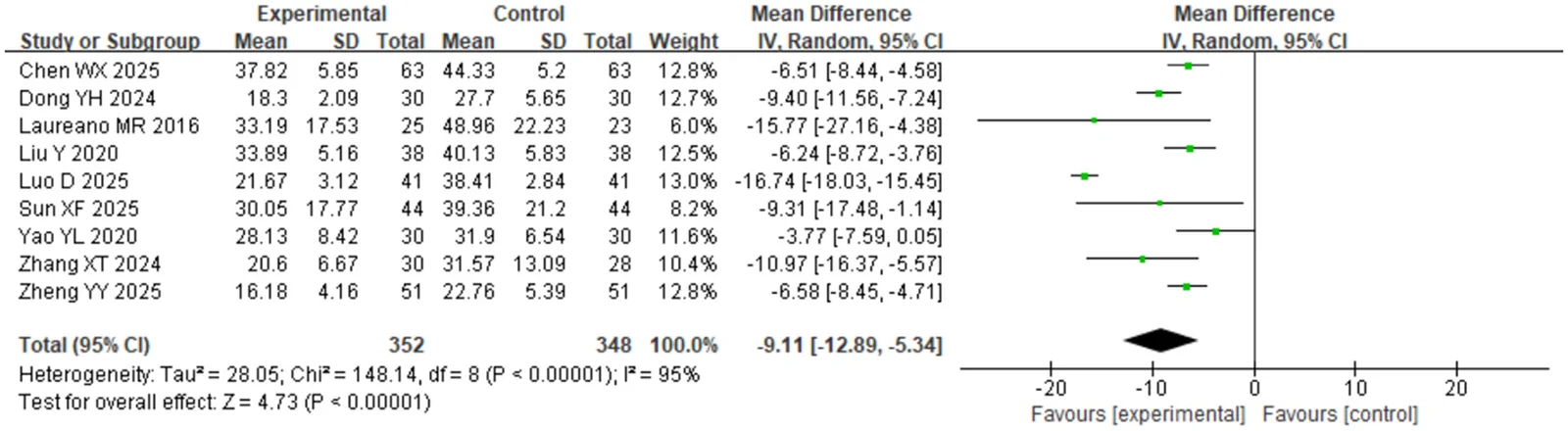
Meta-analysis forest plot of THI scores for acupuncture treatment of tinnitus with anxiety.

##### 3.4.3.1 Subgroup analysis of tinnitus handicap inventory (THI) scores

Based on different control groups, subgroup analysis of THI scores from the nine (15–22, 24) included studies showed statistically significant differences in THI scores between subgroups (*P* < 0.00001). According to a review of the literature, the study by Luo et al. (16) used tuina therapy as the only control intervention; it lacked objective standards and was primarily dependent on the therapist's personal experience, tactile sensitivity, and physical state. The control interventions used in the following eight research (15, 17–22, 24) were more measurable and included Western medication, traditional Chinese medicine, or sham acupuncture. The various control intervention techniques used could be the cause of the heterogeneity. Refe to Figure 6.

**Figure 6:**
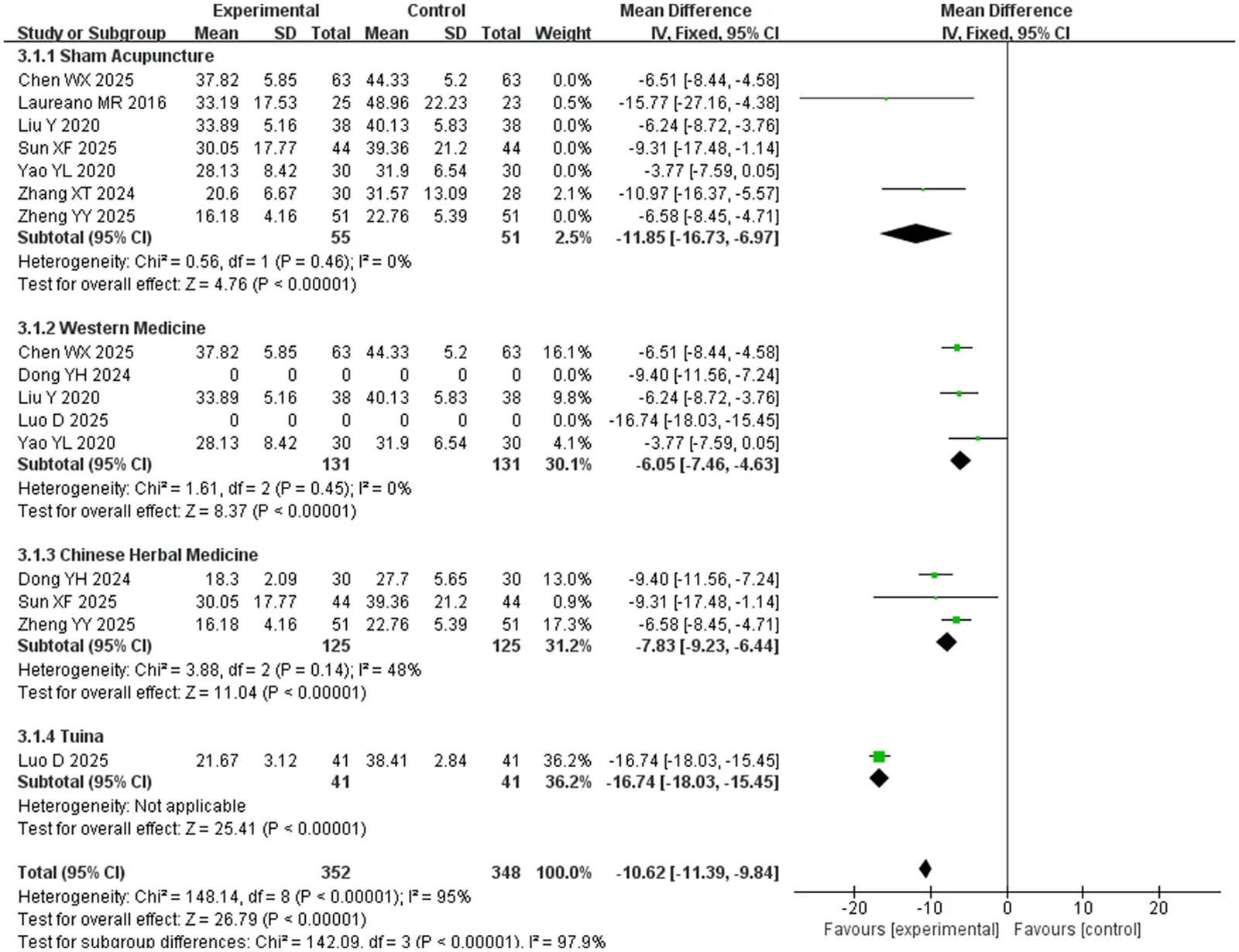
Meta-analysis forest plot for THI score subgroup analysis of acupuncture treatment for tinnitus with anxiety.

#### 3.4.4 Traditional chinese medicine (TCM) syndrome score

Four (16, 17, 19, 21) of the 12 included trials reported TCM syndrome scores before and after treatment. Results demonstrated that the treatment group considerably outperformed the control group in reducing TCM syndrome scores (MD = −0.59, 95% CI: −1.13 to −0.06, *P* = 0.03 < 0.05, *I*^2^ = 96%). Refer to Figure 7. Sensitivity analysis shows the results are robust.

**Figure 7:**
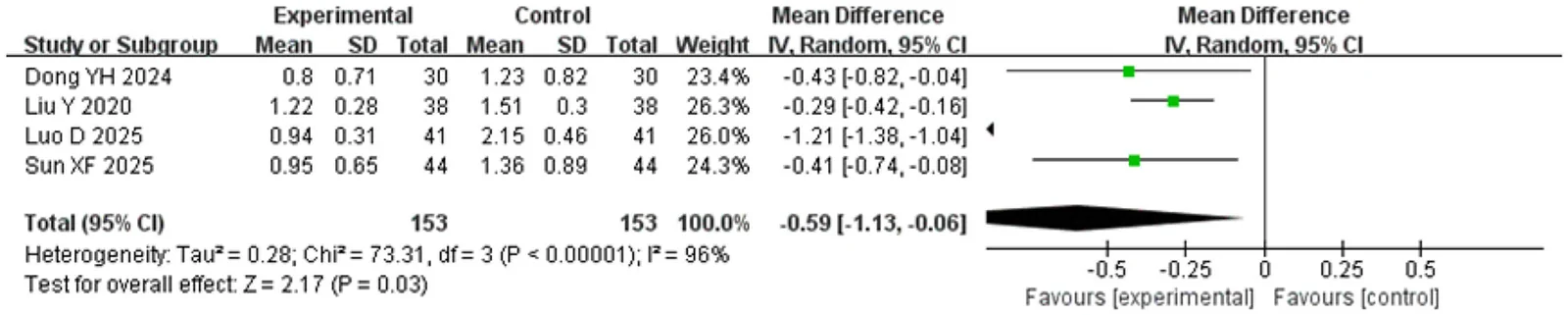
Meta-analysis forest plot of TCM syndrome scores for acupuncture treatment of tinnitus with anxiety.

There was no further heterogeneity (*P* = 0.67, *I*^2^= 0%) once the Luo et al. (16) study was eliminated. Refer to Figure 8. Combined analysis of all research showed that the study by Luo et al. (16) involved patients with longer disease duration (chronic tinnitus phase surpassing 2 years), whereas the remaining three studies (17, 19, 21) focused on subacute (3–6 months) or chronic (≦ 2 years) phases. The disease duration may be connected to the existence of heterogeneity.

**Figure 8:**
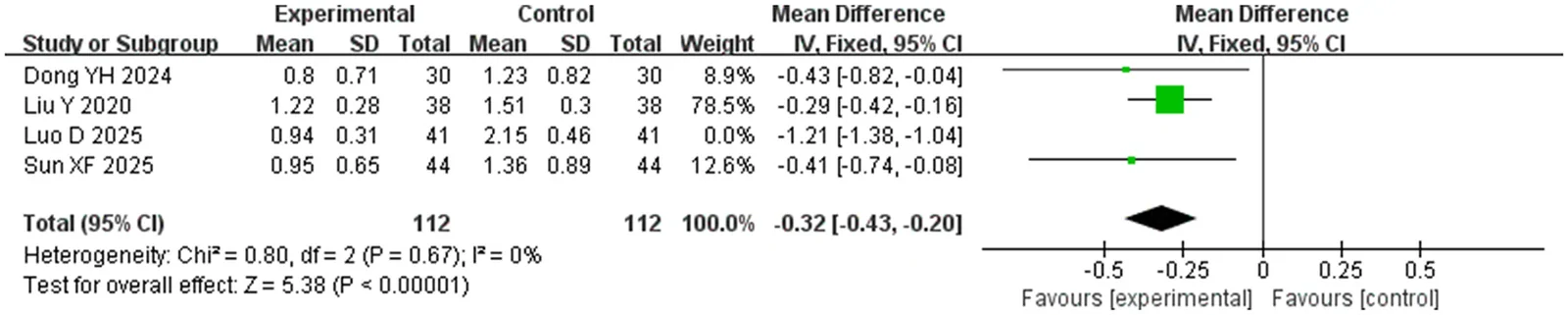
Meta-analysis forest plot of TCM syndrome scores for acupuncture treatment of tinnitus with anxiety (after individual exclusion).

#### 3.4.5 . Hamilton anxiety rating scale (HAMA) scores

Four (14, 20, 22, 24) of the 12 included trials reported HAMA scores before and after treatment. Results demonstrated that the treatment group considerably outperformed the control group in reducing HAMA scores (MD = −2.04, 95% CI: −3.08 to −1.00, *P* = 0.0001 < 0.05, *I*^2^= 82%). Refer to Figure 9. Sensitivity analysis shows the results are robust.

**Figure 9:**
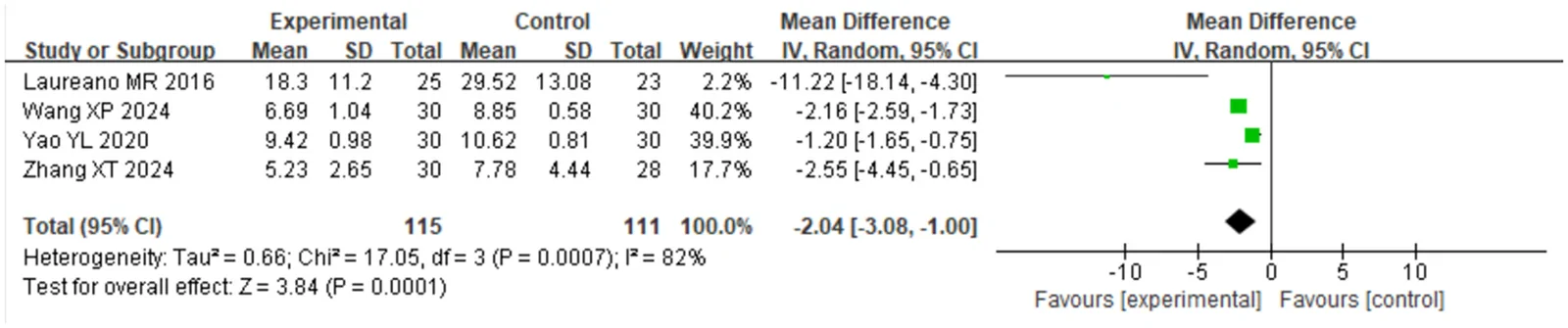
Meta-analysis forest plot of HAMA scores for tinnitus with anxiety treated by acupuncture.

After excluding all studies, heterogeneity remained at least 70%. Among the potential causes of this are: The HAMA is a clinician-rated scale that has to be evaluated by qualified doctors; Compared to other anxiety measures, the HAMA's anxiety state questions are less specific, resulting in more thorough physician assessments and comparatively lower pre- and post-treatment differences.

#### 3.4.6 Self-rating anxiety scale (SAS) scores

Two (15, 17) of the 12 included trials reported SAS scores before and after treatment. Results demonstrated that the treatment group considerably outperformed the control group in reducing SAS scores (MD = −3.50, 95% CI: −6.22 to −0.77, *P* = 0.01 < 0.05, *I*^2^= 78%). Refer to Figure 10.

**Figure 10:**
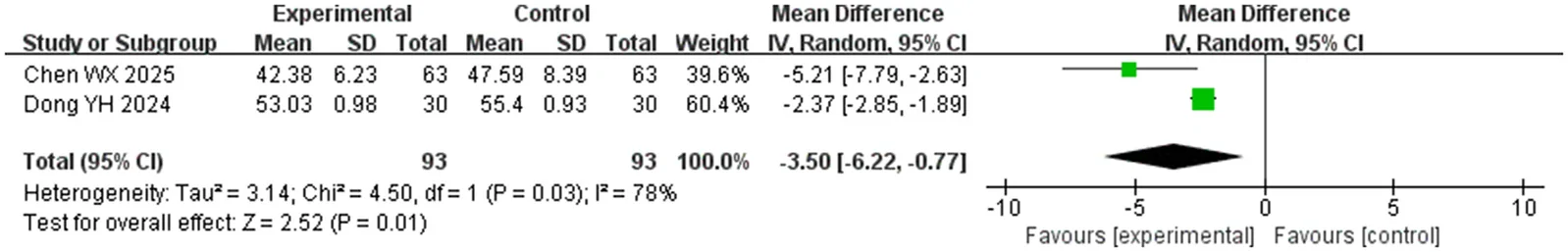
Meta-analysis forest plot of SAS scores for acupuncture treatment of tinnitus with anxiety.

#### 3.4.7 Beck anxiety inventory (BAI) Scores

Two (16, 22) of the 12 included trials reported BAI scores before and after treatment. Results demonstrated that the treatment group considerably outperformed the control group in reducing BAI scores (MD = −8.80, 95% CI: −13.48 to −4.12, *P* = 0.0002 < 0.05, *I*^2^= 66%). Refer to Figure 11.

**Figure 11:**
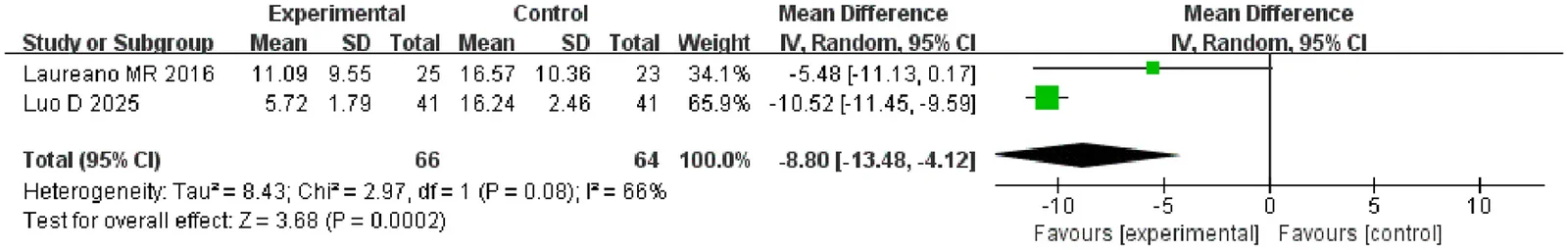
Meta-analysis forest plot of BAI scores for acupuncture treatment of tinnitus with anxiety.

#### 3.4.8 Brain-derived neurotrophic factor (BDNF) levels

Two (13, 24) of the 12 included trials reported BDNF levels before and after treatment. Results demonstrated that the treatment group and the control group did not significantly vary in lowering BDNF levels (MD = −72.27, 95% CI: −312.41 to 167.87, *P* = 0.56 > 0.05, *I*^2^= 80%). Refer to Figure 12.

**Figure 12:**
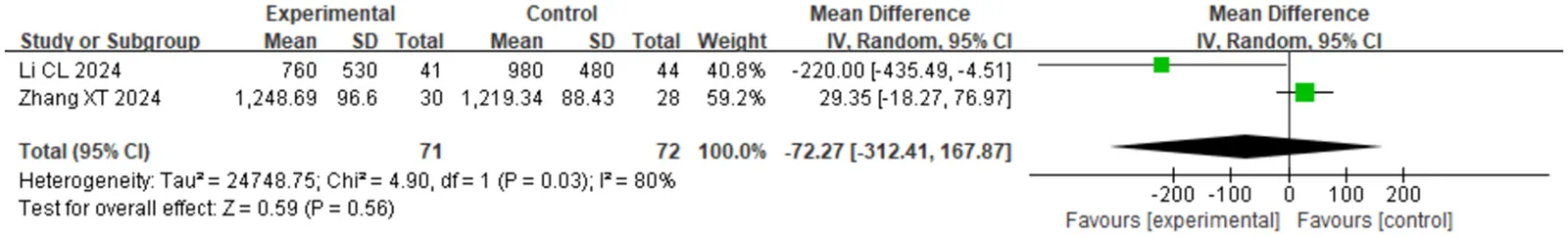
Meta-analysis forest plot of BDNF levels in acupuncture treatment for tinnitus with anxiety.

#### 3.4.9 Serum 5-hydroxytryptamine (5-HT) levels

Two (21, 24) of the 12 included trials reported 5-HT levels before and after treatment. Results demonstrated that the treatment group and the control group did not significantly vary in lowering 5-HT levels (MD = −22.56, 95% CI: −56.12 to 10.99, *P* = 0.19 > 0.05, *I*^2^=99%). Refer to Figure 13.

**Figure 13:**
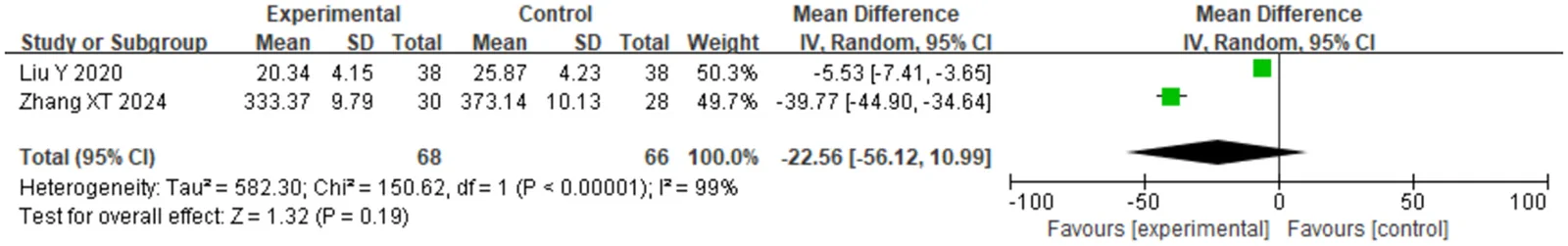
Meta-analysis forest plot of 5-HT levels in tinnitus with anxiety treated by acupuncture.

#### 3.4.10 Serum γ-aminobutyric acid (GABA) levels

Two (21, 24) of the 12 included trials reported GABA levels before and after treatment. Results demonstrated that the treatment group and the control group did not significantly vary in lowering GABA levels (MD = 56.23, 95% CI: −46.50 to 158.96, *P* = 0.28 > 0.05, *I*^2^ =97%). Refer to Figure 14.

**Figure 14:**
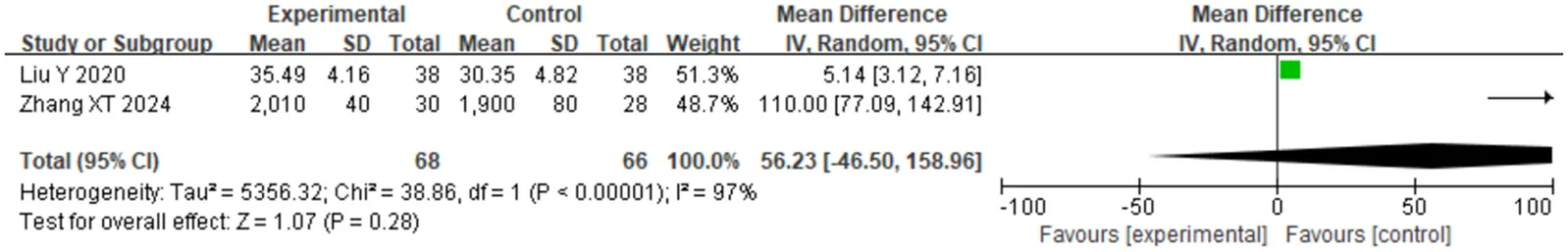
Meta-analysis forest plot of GABA levels in tinnitus with anxiety treated by acupuncture.

### 3.5 Publication bias

A funnel diagram was produced by statistically analyzing the clinical overall effective rate of the included trials using RevMan 5.3 software. A low probability of publication bias was suggested by the data, which displayed a bilaterally symmetrical distribution of the included research. It should be mentioned that this evaluation may contain subjectivity and bias because it was based on firsthand observation. Refer to Figure 15.

**Figure 15:**
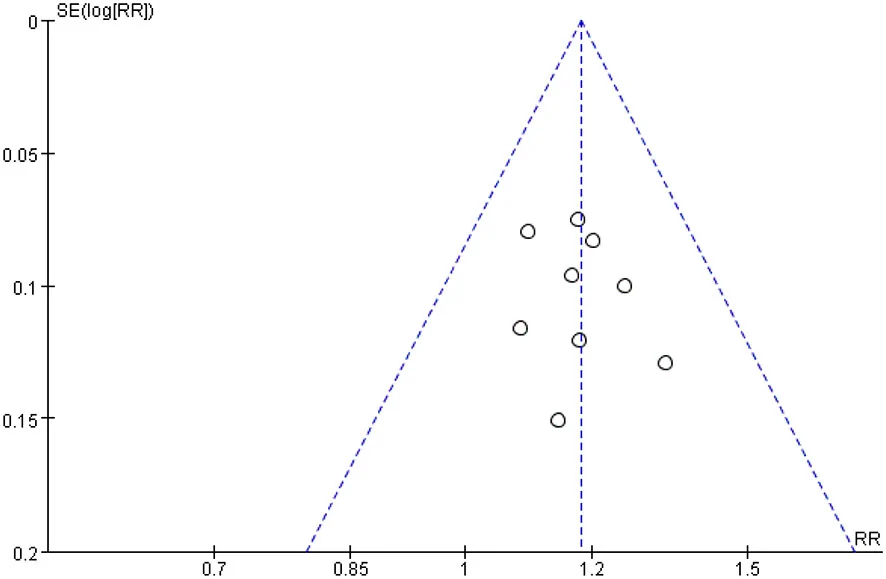
Publication bias risk funnel plot.

### 3.6 Evidence quality assessment

Three pieces of evidence were graded as high quality, one as moderate quality, and six as low quality in this study. Risk of bias (ROB), which is represented by inadequate or inconsistent descriptions of allocation concealment techniques and inexplicable blinding protocols, which is represented by unexplained heterogeneity, and imprecision, which is characterized by excessively wide confidence intervals, were the main causes of this downgrading. As seen in Supplementary Figures 1–10 offer a thorough explanation of these problems for additional analysis.

## 4 Discussion

In otolaryngology, tinnitus is a frequent subjective condition that is difficult to fully cure and has a high recurrence rate. The incidence of tinnitus has been continuously increasing in recent years, with a trend toward younger age groups due to the acceleration of social pace, greater living stresses, and growing healthcare costs. Additionally, a growing number of tinnitus sufferers report concomitant symptoms like melancholy, anxiety, sleeplessness, vertigo, and hearing loss. The most common emotional disorders among them are depression and anxiety (25). Anxiety can present as both a concurrent symptom and a causal element of tinnitus (26). According to research (27, 28) tinnitus severity correlates not with loudness, pitch, or duration, but closely with negative emotions induced by tinnitus (29, 30). In order to assess the effectiveness of acupuncture in treating tinnitus associated with anxiety, this study performed a meta-analysis of 12 randomized controlled trials. According to the main results, acupuncture clearly outperformed control groups (sham acupuncture, Western medication, etc.) in terms of increasing overall efficacy rate, decreasing tinnitus severity (TEQ), easing tinnitus-related disability (THI), and easing anxiety symptoms (HAMA, SAS, BAI). However, there were no statistically significant variations between the groups in the levels of specific neurotransmitters (BDNF, 5-HT, GABA) that may be linked to tinnitus and anxiety.

The eardrum vibrates as a result of sound waves that are collected by the outer ear and sent to it via the external auditory canal. The three ossicles in the middle ear move in unison as a result of this vibration, which also causes the lymph fluid in the inner ear's cochlea to create pressure waves. Different parts of the basilar membrane resonate at different frequencies as a result of these waves. Auditory nerve terminals are activated by bioelectric impulses produced by the bending of hair cell stereocilia. The auditory area in the cerebral cortex then receives these impulses in a stepwise upward direction. The ultimate auditory perception is created following additional integration, analysis, and recognition. Tinnitus may develop as a result of damage at any stage of this process.

Although the medical world is still unsure of the precise mechanism underlying tinnitus, clinical research has shown that the thalamus-auditory cortex-amygdala pathway interprets tinnitus as a negative emotion when it lasts for an extended period of time and develops into chronic tinnitus. The tinnitus signal is further processed, refined, and validated by the route during this process, which makes the amygdala more sensitive to unpleasant emotions. It might be interpreted as a negative emotion via the auditory cortex-amygdala connection. The amygdala's sensitivity to negative emotions is increased during this process as the pathway further cognitively evaluates and confirms the tinnitus signal. A vicious loop of “tinnitus → negative emotion → amplified tinnitus” is thus created (31).

Numerous scientific and clinical studies have shown that tinnitus is tightly associated with psychological processes including emotions and memory, rather than being restricted to the auditory system (32). These days, an increasing number of researchers are observing that tinnitus development is significantly influenced by the limbic system (33). The limbic system is comprised of three main parts: diencephalic structures like the hypothalamus and anterior thalamic nuclei; subcortical parts like the nucleus accumbens and amygdala; and cortical areas like the cingulate gyrus, orbitofrontal cortex, entorhinal cortex, hippocampus, etc. Patients with tinnitus have been discovered to have anomalies in the limbic system, including the amygdala, hippocampus, nucleus accumbens, and frontal lobe, according to several investigations employing contemporary imaging techniques including SPECT, PET, and fMRI (34). Zhang et al. (35) found that in noise-induced tinnitus guinea pigs, besides seeing C-fos markers in the auditory cortex and inferior colliculus, there was also noticeable C-fos expression in the amygdala and hypothalamus. After long-term sodium salicylate injections, Yi et al. (36) used Micro PET to examine central metabolic changes in rats with tinnitus. They discovered high metabolic regions in the inferior colliculus, auditory cortex, hippocampus, amygdala, and cerebellum, offering concrete proof that the limbic system plays a role in tinnitus.

According to recent studies, acupuncture can reduce anxiety-related tinnitus by enhancing peripheral circulation, modifying the limbic system, and controlling changes in brain plasticity (37). Specifically: Activating acupoints like Tinggong (SI19) and Tinghui (GB2) speeds up blood flow, lowers blood viscosity in the inner ear, and restores impaired hearing; Needling locations like Fengchi (GB20) and Wangu (GB12) can minimize cellular damage, lessen negative emotions linked to tinnitus, and lower the mRNA expression of inflammatory cytokines in the hippocampus; By stimulating the motor cortex and paracentral lobules, needling spots like Baihui (GV20) and Yintang (GV24+) can alter the central nervous system and reduce anxiety and depression. The results show that in patients with tinnitus and anxiety, acupuncture treatment is beneficial in improving the total effective rate, TEQ, THI, TCM syndrome scores, HAMA, SAS, and BAI scores. This is consistent with the findings of earlier studies.

According to research (38), acupuncture may increase synaptic plasticity and have anxiolytic and antidepressant effects via activating the BDNF/ERK/mTOR signaling pathway in the lateral habenula of rats. According to literature (39), electroacupuncture can ameliorate stress-induced anxiety-depression-like behaviors in mice by modifying 5-HT receptor levels in the hippocampus. Acupuncture can regulate the Glu/GABA-Gln metabolic cycle by controlling the protein production of GAD65, GAD67, and GS within this cycle, which raises GABA levels and lowers Glu levels, according to another animal study (40). According to the study's findings, acupuncture treatment did not significantly differ in its effectiveness between the treatment group and the control group in terms of raising BDNF, 5-HT, and GABA levels in patients who had anxiety-related tinnitus. This result contradicts the findings of earlier studies. Among the potential causes are: There was not enough statistical power for testing because there were only two studies evaluating these indicators; The regulation of these particular neurotransmitters may not be the primary mechanism of acupuncture's impact; Results cannot be consolidated due to variations in laboratory testing techniques.

The following are the limitations of this study: Potential publishing bias: there was a chance of selective reporting bias that could result in publication bias because the majority of the included studies were in Chinese and were not registered with the China Clinical Trial Registry. Lack of safety analysis and long-term efficacy assessment: it is challenging to ascertain the safety and long-term efficacy of acupuncture for tinnitus with anxiety because the majority of studies did not carry out safety evaluations or follow-up investigations. Incomplete outcome measurements: The Self-Rating Anxiety Scale and other scales were included in the outcome measures, however there is not enough pertinent literature to do a quantitative meta-analysis; Heterogeneity: variations in the selection of acupoints, the application of techniques, the intervention measures, the length of therapy, and the criteria used to evaluate efficacy among the included studies led to an increase in heterogeneity. The origins of heterogeneity were not investigated in this review; future studies will carry out more analysis and validation.

In conclusion, anxiety and tinnitus interact and reinforce one another, and the onset of one condition might cause the other to develop or worsen. Therefore, when treating patients' tinnitus symptoms, clinicians should also concentrate on reducing anxiety in order to further improve therapy efficacy. According to this study, individuals with tinnitus and anxiety may benefit from acupuncture therapy in terms of subjective-reported outcomes (such as TEQ, THI, Traditional Chinese Medicine syndrome scores, HAMA, SAS, and BAI scales). In addition to being a successful alternative and supplemental therapy option, it can lessen the psychological strain on patients. However, in order to strengthen the body of evidence, these conclusions need to be further validated through large-scale, high-quality randomized controlled trials (RCTs) due to the limitations and heterogeneity of existing studies.

## 5 Conclusions

Acupuncture treatment can improve the overall clinical response rate and reduce TEQ, THI, TCM syndrome scores, BAI, HAMA, and SAS scores in patients with tinnitus accompanied by anxiety. However, evidence levels for some outcome measures (BDNF, 5-HT, and GABA) remain low, necessitating further validation through high-quality, large-scale randomized controlled trials.

## Data Availability

The original contributions presented in the study are included in the article/Supplementary material, further inquiries can be directed to the corresponding author.
