# Supplementary material for: Efficacy of acupuncture for tinnitus with anxiety: a systematic review and meta-analysis
Source: Front Neurol. 2026 Sep 22;17:1920000. doi: 10.3389/fneur.2026.1920000 (PMC13638367; doi:10.3389/fneur.2026.1920000)
Supplement: Supplementary file 1 [file Supplementary_file_1.docx]

**GRADE Evidence Quality Assessment：**

Figure 1. GRADE assessment for the Total effective rate

Figure 2. GRADE assessment for the TEQ

Figure 3. GRADE assessment for the THI

Figure 4. GRADE assessment for the Traditional Chinese Medicine Syndrome Scoring

Figure 5. GRADE assessment for the HAMA

Figure 6. GRADE assessment for the SAS

Figure 7. GRADE assessment for the BAI

Figure 8. GRADE assessment for the BDNF

Figure 9. GRADE assessment for the 5-HT

Figure 10. GRADE assessment for the GABA

**Search term：**

**CNKI：**

**VIP：**

**Wangfang：**主题:(耳鸣 OR 神经性耳鸣 OR 原发性耳鸣 OR 主观性耳鸣) and 主题:(焦虑 OR 焦虑状态) and 主题:(针 OR 针刺 OR 针法 OR 针灸)

**SinoMed：**( "针"[常用字段:智能] OR "针刺"[常用字段:智能] OR "针法"[常用字段:智能] OR "针灸"[常用字段:智能]) AND( "焦虑"[常用字段:智能] OR "焦虑状态"[常用字段:智能]) AND( "耳鸣"[常用字段:智能] OR "神经性耳鸣"[常用字段:智能] OR "原发性耳鸣"[常用字段:智能] OR "主观性耳鸣"[常用字段:智能])

**CMAJD：**((标题=*耳鸣 OR 神经性耳鸣 OR 原发性耳鸣 OR 主观性耳鸣*) AND 标题=*焦虑 OR 焦虑状态*) AND 标题=*针 OR 针刺 OR 针法 OR 针灸*

**PubMed：**

**Embase：**

**Cochrane：**

**WOS：**

((TS=(Tinnitus OR Ringing-Buzzing-Tinnitus or Ringing Buzzing Tinnitus or Pulsatile Tinnitus or Tinnitus, Pulsatile or Tinnitus of Vascular Origin or Vascular Origin Tinnitus or Tinnitus, Vascular Origin)) AND TS=(Anxiety OR Angst or Nervousness or Hypervigilance or Social Anxiety or Anxieties, Social or Anxiety, Social or Social Anxieties or Anxiousness)) AND TS=(Acupuncture OR Pharmacopuncture)
